# Strawberry notch 1 safeguards neuronal genome via regulation of *Yeats4* expression

**DOI:** 10.1038/s41420-025-02640-4

**Published:** 2025-07-24

**Authors:** Dai Ihara, Ayano Narumoto, Yukie Kande, Tomoki Hayashi, Yasuaki Ikuno, Manabu Shirai, Masaki Wakabayashi, Ryo Nitta, Hayato Naka-Kaneda, Yu Katsuyama

**Affiliations:** 1 Department of Anatomy, Shiga University of Medical Science, Shiga, Japan; 2 Omics Research Center, National Cerebral and Cardiovascular Center, Osaka, Japan; 3 Division of Structural Medicine and Anatomy, Department of Physiology and Cell Biology, Kobe University Graduate School of Medicine, Kobe, Japan

**Keywords:** Apoptosis, Double-strand DNA breaks

## Abstract

Neurons are subjected to various stresses, including high metabolic demand, physiological activity, and transcriptional regulation, to which their genomic DNA are vulnerable. Genome stability of neurons is essential for proper physiological brain function. Failure in accurate genomic DNA repair can result in abnormal neuronal functions or cell death. Genomic instability has been implicated in increased risks of neurodevelopmental and neurodegenerative disorders. However, the molecular mechanisms underlying neuronal genome stability remain poorly understood. Mutations in the *Strawberry Notch Homolog 1* (*SBNO1*) have been suggested to contribute to these disorders. Here, we investigated the molecular mechanisms underlying histological abnormalities observed in the cortex of *Sbno1* knockout (KO) mice. Comprehensive gene expression analysis revealed that *Sbno1* KO affects the expression of genes related to cell survival, consistent with the increased apoptosis observed in *Sbno1* KO cortices. Among the genes downregulated in *Sbno1* KO, we focused on *Yeats4*. Overexpression of Yeats4 rescued the accumulation of genomic DNA damage and cell death caused by *Sbno1* deletion. These findings suggest that Sbno1 is critical in safeguarding the neuronal genome, at least in part, via regulating *Yeats4* expression.

## Introduction

Neurons in the brain are primarily generated during the embryonic and fetal stages. After birth, neural stem cells in specific brain regions can generate only a limited range of neuron types [1]. However, neuronal genome is particularly susceptible to damage and is easily destabilized by stresses. Neurons sustain a high metabolic demand to effectively transmit information, consuming approximately 25% of the body’s oxygen needs [2, 3]. When the immediate early genes (IEGs) are upregulated in response to physiological activity of neurons, DNA double-strand breaks (DSBs) alleviates topological stress allowing enhance-promoter interactions. Thus, neurons have poor regenerative capacity, despite their genomic DNA is highly vulnerable.

In proliferative cells, DSBs are precisely repaired through homologous recombination, utilizing homologous chromosomes as templates [4–6]. In contrast, non-proliferative neurons predominantly rely on non-homologous end joining (NHEJ) [7], a more error-prone DNA repair mechanism that often results in mutations. Extensive genomic research has established a positive correlation between the accumulation of de novo mutations and the onset of neurological disorders [8, 9]. However, the molecular mechanism of long-term neuronal genome integrity is not fully understood [10].

Human genome studies have suggested the involvement of *SBNO1* gene mutations in neurodevelopmental diseases [11–16]. However, the molecular function of SBNO1 remains underexplored. We immunohistochemically showed that Sbno1 is specifically expressed in neurons in the brain [17, 18] and experimentally demonstrated that *Sbno1* deficient cortical neurons exhibit impaired neurite growth [17]. In this study, we investigated molecular phenotypes of the *Sbno1* KO cortex and found sparse apoptosis of cortical neurons, along with significant downregulation of the *Yeats4* gene. Overexpressing Yeats4 ameliorated genomic DNA degeneration in *Sbno1* deficient neurons. These experimental results revealed that Sbno1 is essential for safeguarding the neuronal genome.

## Results

### Increase of apoptosis in *Sbno1*-deficient cortices

**Fig. 1:**
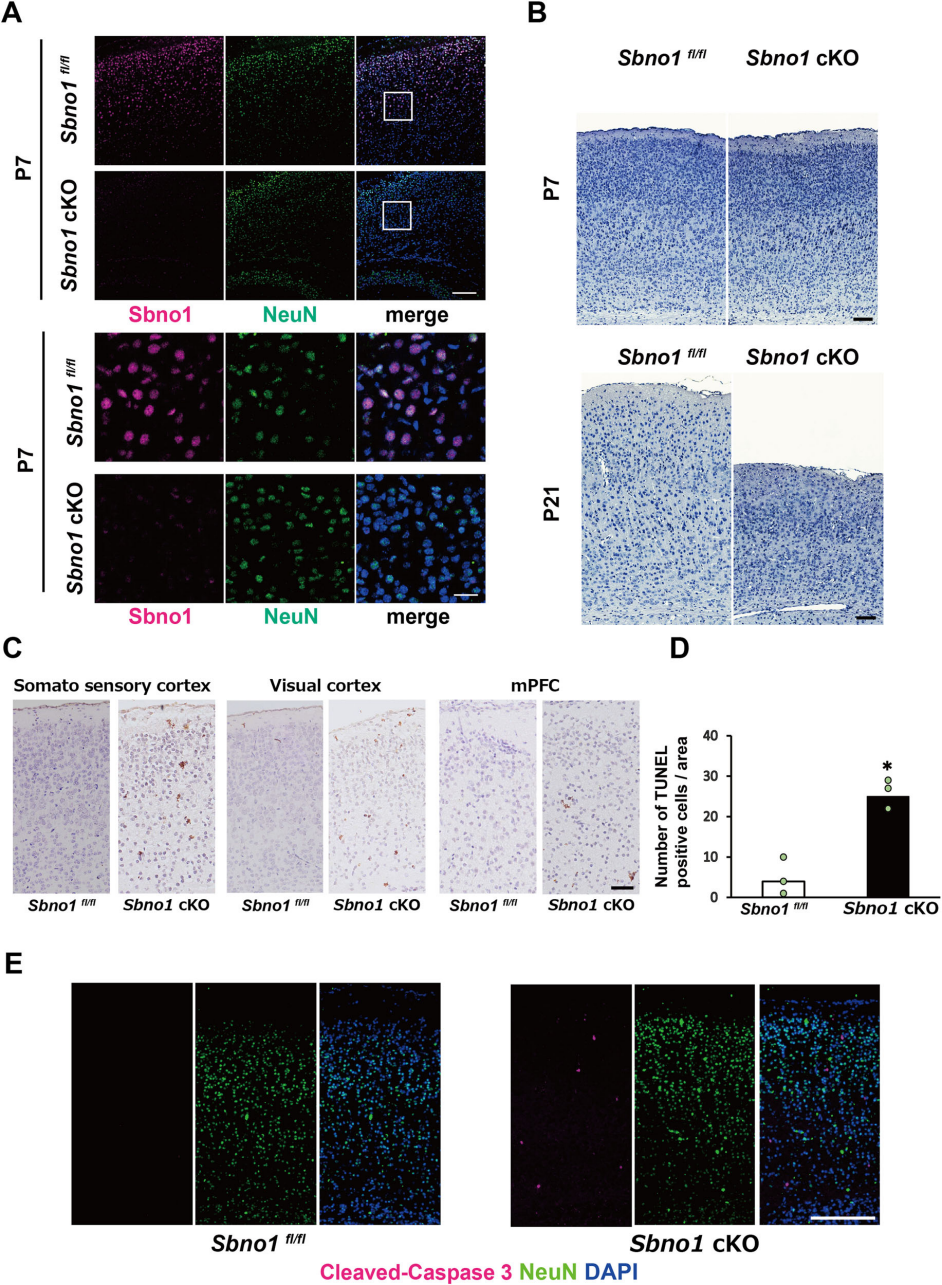
Neurodegeneration in the *Sbno1* deficient cortex. **A** Sbno1 is expressed in cortical neurons identified by NeuN expression in the control (*Sbno1*^*fl/fl*^) cortex. Sbno1 immunoreactivity was lost in *Sbno1*^*fl/fl*^*;Nex-Cre* conditional knockout (*Sbno1* cKO) cortex. Immunostaining images of the cortex at postnatal day (P)7. Scale bar = 100 μm. The lower panels show high-magnification images of the region indicated by squares in upper panels. Scale bar = 25 μm. **B** Nissl staining images of primary somatosensory cortices of control and *Sbno1* cKO mice at P7 (upper) and P21 (lower). Scale bar = 100 μm. **C** TUNEL staining images of control and *Sbno1* cKO cortices in the primary somatosensory, visual, and prefrontal cortices. Brown dots are TUNEL positive cells. The sections were counter stained by hematoxylin (blue). Scale bar = 100 μm. **D** Number of TUNEL-positive cells in each cortical area in a μm width. Data are mean values per 1 mm² for each cortical area from three brains. *p < 0.05. **E** Immunostaining images detecting CC-3 in P7 control and *Sbno1*cKO cortices. Scale bar = 100 μm.

To investigate Sbno1 function in postmitotic neurons, we crossed floxed *Sbno1* transgenic mice with *Nex-Cre* driver mice, in which Cre recombinase is specifically expressed in postmitotic neurons [19] (Fig. S1A). Loss of Sbno1 protein in the neuron-specific *Sbno1* knockout (*Sbno1*^*floxflox*^*; Nex-Cre; Sbno1* cKO) mice was immunohistochemically confirmed (Fig. 1A). Although no significant histological differences were observed between sibling controls (*Sbno1*^*flox/flox*^) and *Sbno1* cKO mice at postnatal day 7 (P7), the *Sbno1* cKO cortices were significantly thinner than that of control at P21 (Figs. 1B and S1B).

Furthermore, we investigated whether cortical thinning was attributable to neuronal cell death using terminal deoxynucleotidyl transferase dUTP nick-end labeling (TUNEL) staining. TUNEL-positive cells were sporadically observed in *Sbno1* cKO somatosensory, visual, and medial prefrontal cortices, but were scarce in the control mice at P7 (Fig. 1C, D). We also observed increase of cleaved caspase-3 (CC-3) expressing cells in the *Sbno1* cKO cortex. Because too few cells undergoing apoptosis to account for cortical thinning by *Sbno1* cKO (Fig. 1E), it is likely that apoptosis is not directly regulated by *Sbno1*.

### Differentially expressed genes in *Sbno1*-deficient cortices

**Fig. 2:**
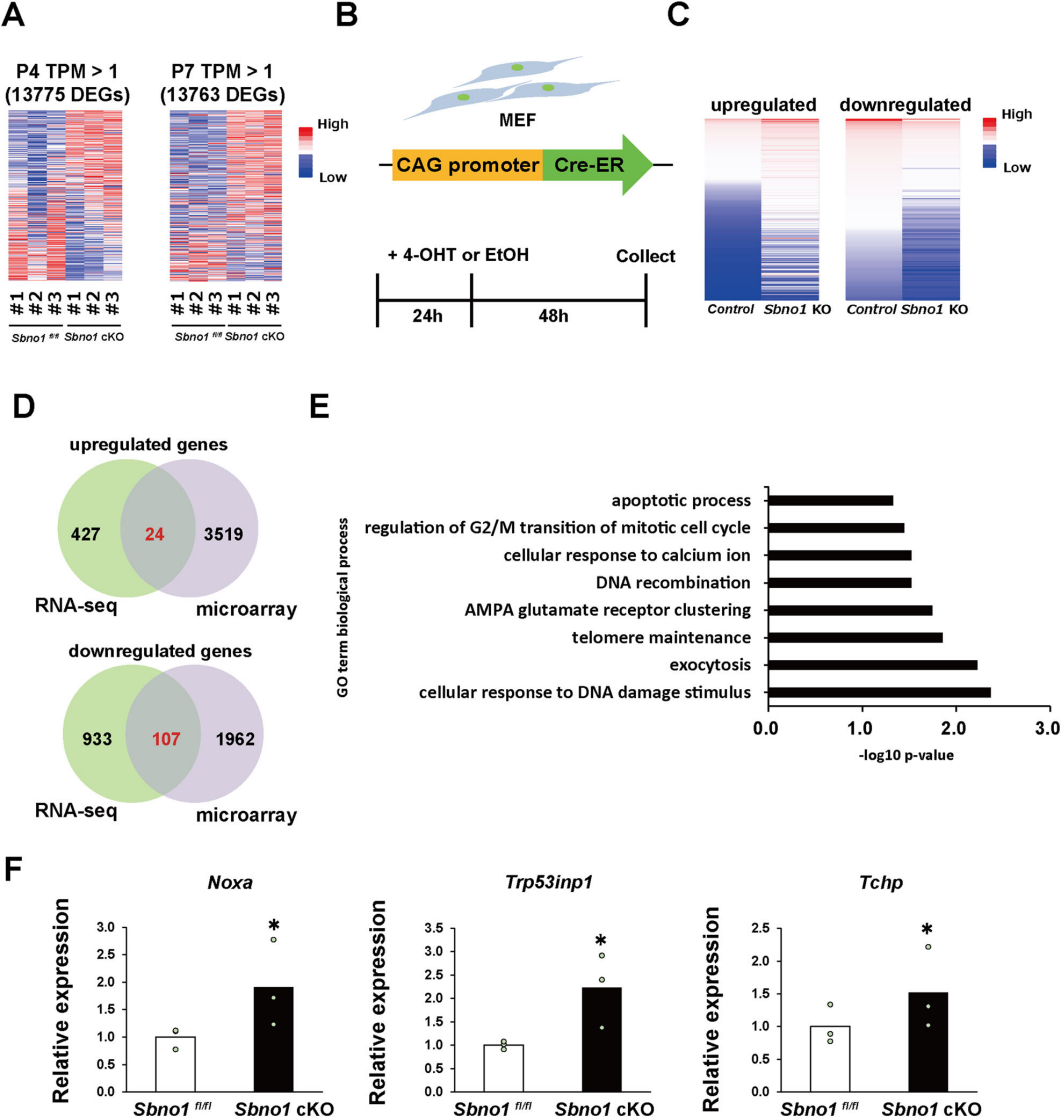
Comprehensive analyses of differentially expressed genes caused by *Sbno1* knockouts. **A** Heatmaps visualizing results of RNA-seq comparing gene expression between control and *Sbno1* cKO cortices at P4 and P7. Triplicate biological samples for each genotype were examined. *n* = 3. **B** Schematic diagram of the CAG-CreER construct. Upon tamoxifen treatment, exon 3 of *Sbno1* gene is excised, resulting in a frameshift producing non-functional protein. **C** Heatmaps visualizing results of microarray comparing control and *Sbno1* cKO mouse embryonic fibroblasts. **D** Venn diagrams depicting upregulated and downregulated genes shared by *Sbno1* cKO in the cortices and embryonic fibroblasts. **E** GO enrichment analysis of overlapping DEGs, with biological processes determined by DAVID. **F** Relative gene expression levels of apoptotic factors in control and *Sbno1* cKO cortices at P7 examined by qPCR; *n* = 3 *p* < 0.05.

Gene expression profiles were compared between the *Sbno1*^*fl/fl*^ (control) and *Sbno1* cKO cortices at P4 and P7 employing RNA sequencing (RNA-seq). Differentially expressed genes (DEGs) were identified based on an absolute TPM value > 1 as the threshold (Fig. 2A). In the P4 cortices, 726 genes were upregulated, and 1180 were downregulated in *Sbno1* cKO. Moreover, 427 upregulated genes and 993 downregulated genes were commonly observed in P4 and P7 cortices (Fig. S2A). Gene ontology (GO) analysis for biological processes revealed that synaptic function-related genes were enriched DEGs (Fig. S2B). According to clustering by Kyoto Encyclopedia of Genes and Genomes (KEGG) pathway, DEGs were associated with neurotransmitters and synapse formation (Fig. S2C). These results are consistent with our previous report that *Sbno1* deficiency reduces neurite growth in cortical neurons [17].

It is possible that sporadic apoptosis in the *Sbno1* cKO cortices is a secondary effect of reduced neuronal connections by altered gene expression of synapse-related molecules. As the *Sbno1* cKO cortex exhibits neurodegeneration (Fig. 1B), it is also possible that basic cellular activities are affected by Sbno1 deficiency and secondarily altering neuronal genes expression. Since a gene expression database (https://www.ebi.ac.uk) indicated that *Sbno1* is expressed in mouse embryonic fibroblasts (MEFs), we examined the effects of Sbno1 deficiency on gene expression in MEFs employing a tamoxifen-inducible gene knockout system. MEFs were collected from the E13.5 *Sbno1*^*floxflox*^*; CreEsr* and *Sbno1*^*floxflox*^ embryos. Cultured MEFs were treated with tamoxifen for 24 h and collected 48 h after medium change for microarray analysis (Fig. 2B). Microarray analysis confirmed that Sbno1 expression was markedly downregulated by tamoxifen treatment (Fig. S2D). We observed 3543 upregulated and 2132 downregulated genes in *Sbno1* KO MEFs (Fig. 2C). Overlap analysis between DEGs comparing control and *Sbno1* KO cortices and DEGs comparing control and *Sbno1* KO MEFs indicated 24 upregulated and 107 downregulated genes shared by *Sbno1* deficiencies (Fig. 2D). GO analysis of these 131 genes revealed enrichment in the apoptotic process (Fig. 2E), consistent with the increased apoptosis in *Sbno1* cKO cortices. To validate our comprehensive gene expression analyses, we quantitatively validated altered expression of significantly upregulated genes—*Noxa* (*Pmaip1*), *Trp53inp1*, and *Tchp*—which are associated with the “apoptotic process” GO term by qPCR. As expected, increased expression of these apoptosis-promoting factors was observed in *Sbno1* cKO cortices (Fig. 2F).

### *Yeats4* is a representative downstream gene of Sbno1

**Fig. 3:**
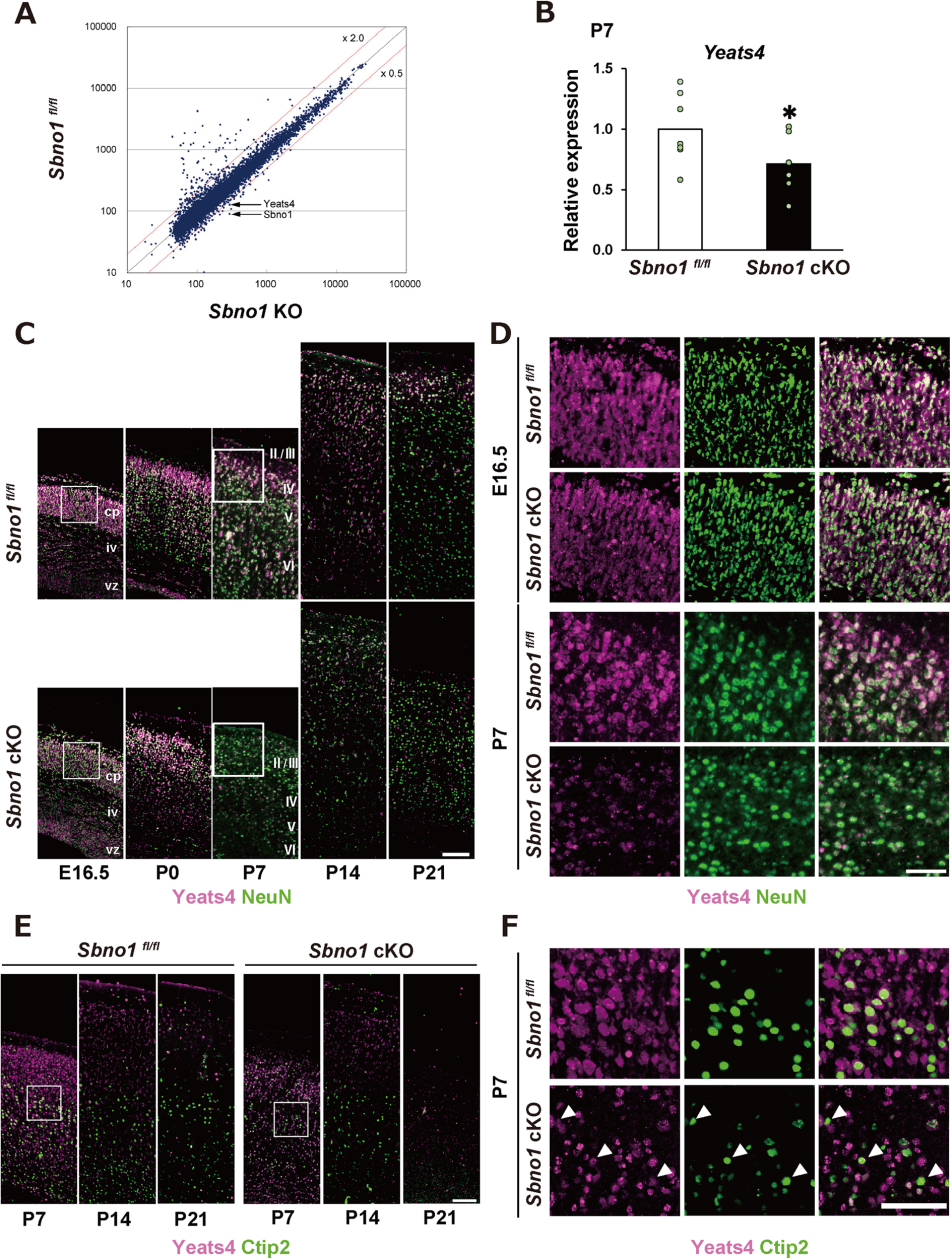
Yeats4 is regulated and expressed in neurons. **A** A scatterplot visualizing results of microarray comparing control and *Sbno1* cKO. The dot highlighted is *Yeats4*, which is the most downregulated gene by *Sbno1* cKO among genes encoding for nuclear factors. **B** Yeats4 expression levels in control and *Sbno1* cKO cerebral cortex validated by qPCR. *n* = 7 *p* < 0.05. **C** Immunostaining images showing expression of Yeats4 and NeuN in the cerebral cortex comparing control and *Sbno1 cKO* cortices at P7 and P21. Scale bar = 100 μm; cp cortical plate, iz intermediate zone, vz ventricular zone. **D** High magnification images of regions indicated by white squares in (**C**). Scale bar: 50 μm. **E** Layer 5 neurons were identified by dense expression of Ctip2 (green). Expression level of Yeats4 (magenta) in layer 5 cortical neurons, which are marked by dense expression of Ctip2 (green) is compared between control and *Sbno1 cKO* at P7, P14, and P21. Scale bar = 100 μm. **F** High magnification images of the region indicated by white square in (**E**). The white arrowheads indicate Ctip2-positive projection neurons. Scale bar = 50 μm.

*Yeats4* was the most significantly downregulated gene in *Sbno1-*deficient MEFs (Fig. 3A), and *Yeats4* expression was significantly reduced in the *Sbno1* cKO cortices (Fig. 3B). Yeats4, also known as GAS41, belongs to a protein family characterized by a conserved domain (Yeats domain), which binds to acetyl-lysine protein modifications [20, 21]. Yeats4 is a component of the NuA4 complex, which is involved in DNA repair [22, 23], and *Yeats4* deletion in tumor cells reduces the efficiency of DNA repair [24]. However, the expression pattern and molecular function of Yeats4 in the brain has not been examined. We immunohistochemically detected Yeats4 during brain development and found that Yeats4 is strongly expressed in cortical neurons (Fig. 3C). In *Sbno1* cKO cortices, Yeats4 expression was slightly reduced at the embryonic stage, and it significantly decreased at P7 and later stages compared with that in the control cortices (Fig. 3C, D). The growth of projection fibers from the cerebral cortex originating from layer 5 projection neurons was impaired in *Sbno1* cKO mice [17], and apoptotic cells were more frequent in layer 5 of *Sbno1* cKO cortices (Fig. S3). Consistent with this, Yeats4 expression in layer 5 projection neurons marked by Ctip2 expression was prominently reduced in the *Sbno1* cKO motor cortex (Fig. 3E, F), suggesting a link between reduction of Yeats4 expression, apoptosis, and neurite growth by Sbno1 deficiency.

### Sbno1 involvement in *Yeats4* transcription

**Fig. 4:**
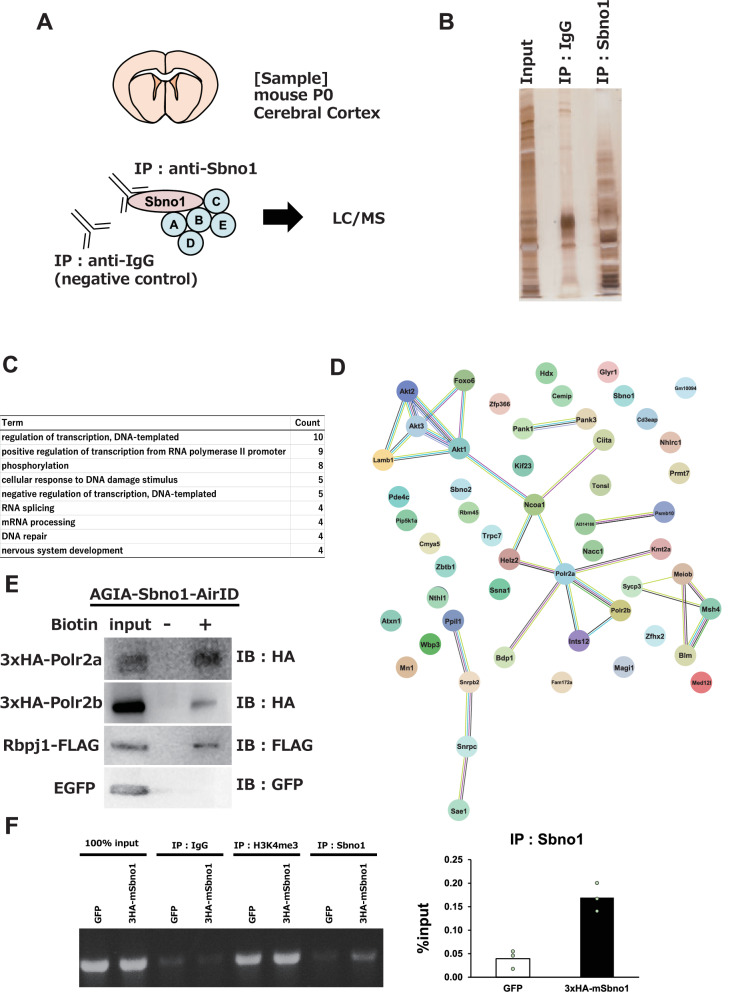
Identification of endogenous Sbno1 interactors in the cerebral cortex. **A** Schematic illustration of the strategy to identify Sbno1-interacting proteins by immunoprecipitation and NanoLC-MS/MS. **B** A Silver staining of SDS-PAGE loaded total protein collected from the cerebral cortex, protein extract immunoprecipitated by anti-IgG antibody, and protein extract immunoprecipitated by anti-Sbno1 antibody. **C** GO enrichment analysis of Sbno1 interactors identified by NanoLC-MS/MS. GO biological processes were analyzed using DAVID. **D** Protein–protein interaction network predicted by STRING. Differently colored lines represent seven types of basis to predict associations: Light blue, pink, green, blue, pale blue, yellow, and black indicates associations predicted from curated databases, experimental determination, gene neighborhood, gene co-occurrence, protein homology, text mining, and co-expression, respectively. **E** Validation of interaction between Sbno1 and RNA polymerase II subunits by proximity biotinylation assay (AirID). The cell cultures overexpressing AirID-Sbno1 and Polr2a, Polr2b, Rbpj, or EGFP were administrated with (+) or without (−) biotin. **F** Left: a band image of qPCR results of CUT&RUN assay suggesting binding of Sbno1 to *Yeats4* promoter. Right: a graph showing relative intensity of bands quantified and statistically compared results of GFP- (control) and Sbno1-overexpression experiments. *n* = 3 *p* < 0.05.

Sbno1 is implicated in the transcription regulation of the Yeats4 gene, yet the precise mechanisms through which Sbno1 influences Yeats4 expression remain elusive. To elucidate potential interactions, we carried out immunoprecipitation (IP) of proteins from an extract of the P0 cerebral cortex using an anti-Sbno1 antibody (α-Sbno1) (Fig. 4A). Both the crude α-Sbno1 IP and control IP extracts were electrophoresed on an acrylamide gel and visualized via silver staining (Fig. 4B). Distinct band patterns between the loaded input, control IP, and α-Sbno1 IP suggested selective enrichment of proteins interacting with Sbno1 by α-Sbno1 IP (Fig. 4B). Subsequently, we analyzed proteins immunoprecipitated by α-Sbno1 utilizing Nano Liquid Chromatography-Mass Spectrometry (NanoLC–MS/MS), and identified 138 potential Sbno1 interactors. Given that Sbno1 is immunohistochemically detected in the nucleus of cortical neurons (Fig. 1A), we focused on 53 proteins predicted to localize in the cell nucleus (Fig. 4C). Proteins involved in transcription were predominant among the Sbno1 interactors. STRING analysis reported that Sbno1 associates with RNA polymerase II subunits, Polr2a and Polr2b, suggesting that Sbno1 play a role in transcription (Fig. 4D). We employed the ancestral BioA for proximity-dependent biotin identification (AirID) assay [25] to validate the mass spectrometry results, and confirmed interaction of Sbno1 with RNA polymerase II subunits (Fig. 4E). As Sbno1 is a member of the helicase family, which interacts with nucleotide chains, we examined its binding to the *Yeats4* promoter region using a cleavage under targets and release using nuclease (CUT&RUN) assay. Our result showed an increased qPCR signal for the *Yeats4* promoter in samples immunoprecipitated with α-Sbno1, suggesting a direct binding of Sbno1 to the promoter (Fig. 4F). These findings indicate that Sbno1 act directly on the *Yeats4* promoter to regulate its transcription.

### Yeats4 rescues DNA damage and apoptosis caused by Sbno1 deficiency

**Fig. 5:**
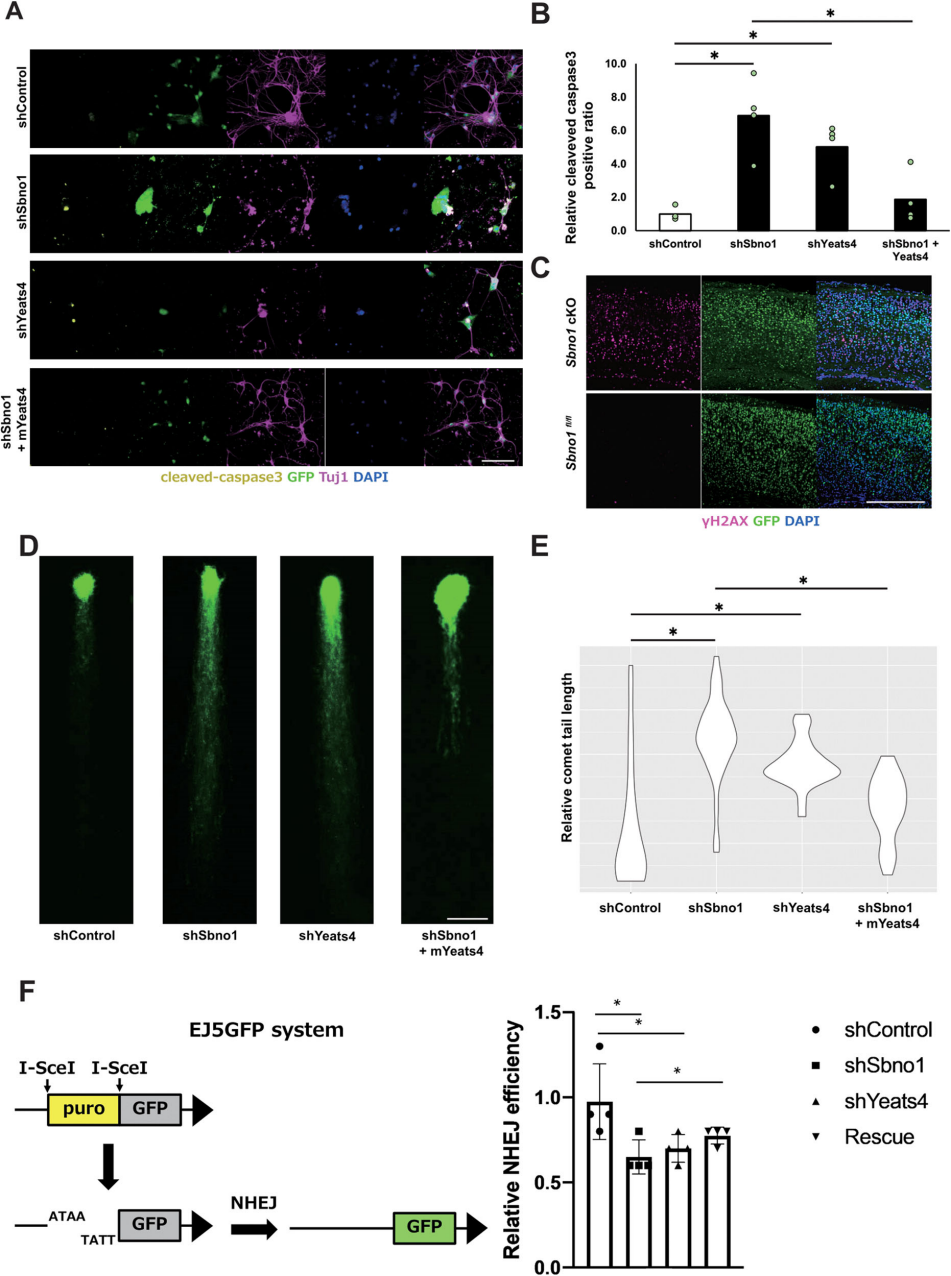
Yeats4 functions downstream of Sbno1 in neuronal survival. **A** Expression of cleaved caspase-3 (CC-3; yellow) was examined in primary neuron cultures transfected with shRNA-control, shRNA-Sbno1, shRNA-Yeats4, or sh-Sbno1 and Yeats4 overexpression plasmids. Neurons were identified by Tuj1 expression (magenta) and transfected cells were marked by GFP expression (green). Scale bar = 100 μm. **B** Ratios of CC-3-positive cells in GFP expressing cells relative to that of the control experiment (shControl). Five fields were randomly selected for each culture, and data from three cultures were averaged. Statistical significance was determined by two-tailed Welch’s *t*-tests. *n* = 4 *p* < 0.05. **C** γH2AX expression was prominently increased in the *Sbno1* cKO cortex than that in control cortex at P7 Scale bar = 250 μm. **D** Comet assay of neurons transfected with shRNA-control, shRNA-Sbno1, shRNA-Yeats4, or sh-Sbno1 and Yeats4 overexpression plasmids. Scale bar = 50 μm. **E** Relative comet tail lengths are shown by violin plot. Statistical significance was determined by two-tailed Welch’s *t*-tests. shControl: *n* = 26, shSbno1: *n* = 31, shYeats4: *n* = 25, shSbno1 + mYeats4: *n* = 24, *p* < 0.05. **F** The right panel is schematic explanation of pEJ5 reporter assay. DSBs are induced by overexpression of the restriction enzyme (I-SceI), and GFP is expressed when DSBs are repaired by NHEJ. The left panel shows quantitative comparison of GFP expression detected by flow cytometry. *n* = 4 *p* < 0.05.

To explore the functional relationship between Sbno1 and Yeats4 in cortical neurons, we constructed gene knockdown (KD) plasmids using three different short hairpin RNA (shRNA) sequences targeting each gene. Sbno1 KD by transfection of shRNA-KD construct (Fig. S4A–C) into primary culture of the cortical neurons reduced *Yeats4* expression, suggesting cell autonomous regulation of *Yeats4* by Sbno1 in neurons (Fig. S4D). Although Sbno1 deficiency downregulates *Yeats4* expression, the molecular mechanism by which Sbno1 influences Yeats4 expression remains unclear. To explore the functional relationship between Sbno1 and Yeats4 in cortical neurons, we examined the roles of these proteins in primary cultured cortical neurons. We observed increased apoptosis in cortical neurons by Sbno1 or Yeats4 KD at comparable levels (Fig. 5A, B). To assess if the enhanced neuronal cell death by Sbno1 KD occurred through Yeats4, we conducted functional rescue experiments. Overexpression of Yeats4 in Sbno1 KD neurons significantly restored neuronal cell death.

Yeats4 is required for DSB repair [24]. Hence, neuronal cell death by *Sbno1* KD or *Sbno1* cKO may be due to the accumulation of DSBs. Immunostaining for γH2AX was performed to examine the accumulation of DNA damage. Although no accumulation of DSBs was observed at E16.5 and P0 in *Sbno1* cKO (Fig. S5A), accumulation of DSBs was detected at P7, coinciding with the marked reduction of Yeats4 expression in *Sbno1* cKO (Fig. 5C), indicating that Yeats4 likely contributes to genomic stability downstream of Sbno1. Furthermore, we employed the comet assay to explore DNA damage under Sbno1 or Yeats4 KD. Both Sbno1 KD and Yeats4 KD neurons extended the comet tail indicating increased DNA damage, whereas overexpression of Yeats4 in Sbno1 KD neurons shortened the comet tail (Fig. 5D, E), suggesting that Yeats4 restores genomic stability compromised by Sbno1 KD.

We also examined the hierarchical relationship between Sbno1 and Yeats4 in regulating apoptosis related gene expression in *Sbno1* cKO cortices. *Noxa* expression was elevated by both Sbno1 and Yeats4 KD (Fig. S5B), and Yeats4 overexpression partially suppressed *Noxa* expression increased in Sbno1 KD neurons. Both Sbno1 and Yeats4 KD increased Trp53inp1 expression, but Yeats4 overexpression did not reversed change of Trp53inp1 expression by Sbno1 KD. Although *Tchp* expression was upregulated in *Sbno1* cKO cortices, it was noｔ affected by KD experiments. Thus, it is likely that these 3 genes are regulated by different mechanisms involving Sbno1.

Since neurons are in the G0 phase of the cell cycle, NHEJ is the primary pathway for repairing DNA DSBs [7] (Fig. 5F). Therefore, NHEJ efficiency was assessed employing EJ5 reporter [26]. A reduction in NHEJ efficiency was observed by Sbno1 KD. Similarly, as reported in previous studies, Yeats4 KD also led to decreased NHEJ efficiency (Fig. 5F). Notably, overexpression of Sbno1 restored NHEJ efficiency even under Yeats4 knockdown conditions (Fig. 5F). These findings suggest that the Sbno1–Yeats4 axis contributes to neuronal survival by promoting NHEJ.

## Discussion

**Fig. 6:**
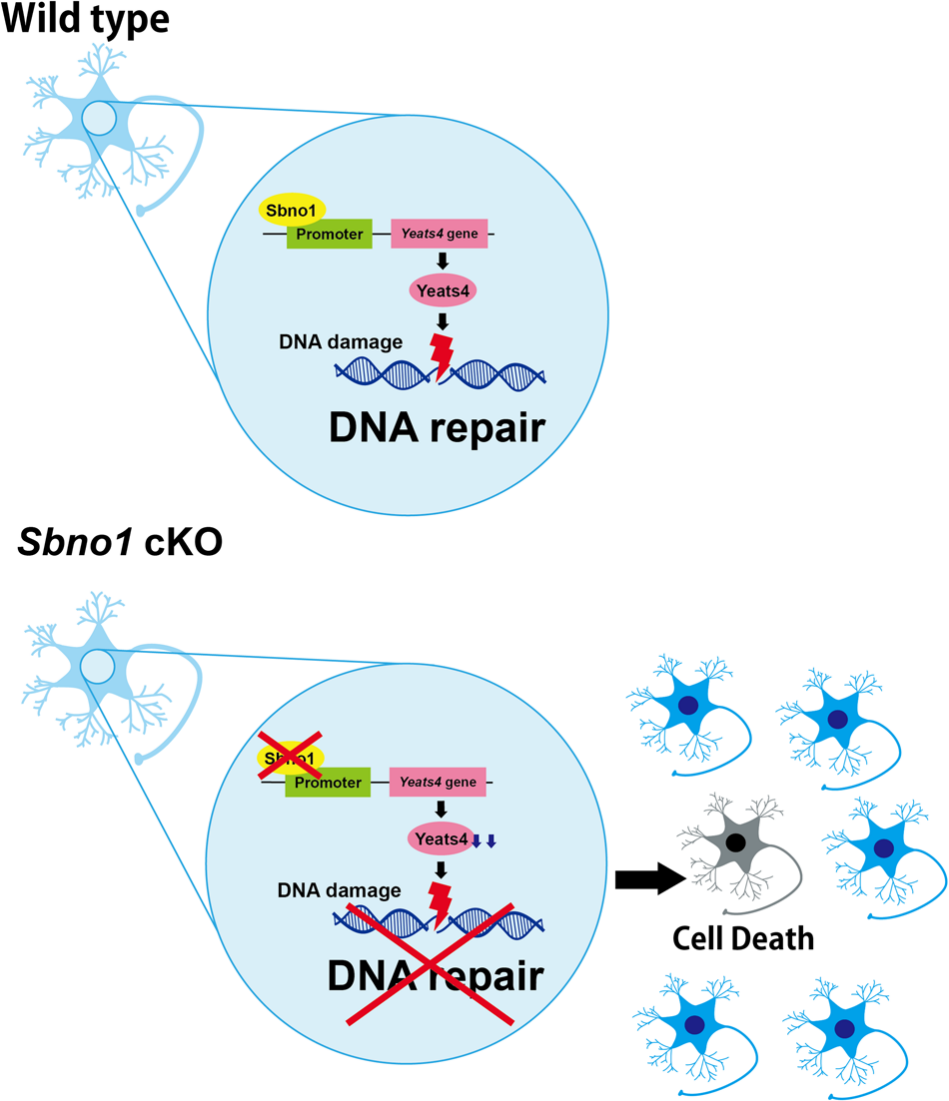
Schematic summary of experimental results in this study suggesting function of Sbno1. Sbno1 regulates Yeats4 transcription in neurons. Yeats4 contributes NHEJ to repair genome DNA. Sbno1-deficiency decreases Yeats4 expression and impairs NHEJ, leading to DNA damage accumulation, and subsequently causes sporadic apoptosis of neurons.

In this study, we elucidated a novel molecular mechanism protecting genome of the neurons. We demonstrated that *Sbno1* deficiency leads to marked reductions of Yeats4 expression in the cortex and primary culture of neurons, and fragmentation of genomic DNA in neurons due to Sbno1 KD. We showed that apoptosis and genomic degeneration by Sbno1 KD were ameliorated by Yeats4 overexpression. Given that Yeats4 plays a role in the NHEJ [24], Sbno1 is a novel factor safeguarding neuronal genomic DNA through regulation of *Yeats4* expression (Fig. 6).

Previous functional analyses during early developmental stages showed that a straight knockout of the *Sbno1* gene in mice resulted in lethality due to significant apoptosis during early cell cleavage stages [27]. In contrast, Sbno1 KD using a morpholino oligonucleotide did not induce cell death in mouse spermatogonia [28]. Within the cerebral cortex, Sbno1 is expressed in all neurons and prominent in layer 5 neurons [18]and more apoptotic cells and reduction of Yeats4 expression were observed in layer 5 in *Sbno1* cKO (Figs. 3F and S3). γH2AX expression was homogenously observed throughout the *Sbno1* cKO cortex (Fig. 5C). These suggest that function of Sbno1 is context dependent, such as developmental stage and neuronal subtypes.

Yeats4 is integral to the Npas4-NuA4 complex [29, 30]. When neurons receive synaptic stimulations, IEGs are rapidly induced [31, 32]. Neuronal stimulation induces DSBs to bring the promoter and enhancer into proximity to activate IEGs expression, whereas Npas4-NuA4 complex is involved in DSB repair, suggesting that DSBs implicates in IEGs activation and one of IEGs is involved in DBS repair to preserve genome integrity [22]. The upregulation of Yeats4 promotes DNA damage repair and prevents cell death, while its downregulation reduces NHEJ efficiency [24]. Yeats4 is also a major component of the SRCAP complex that converts H2A to H2Z in the histone complex. This conversion of histone isoforms is a landmark upon DSBs to promote DNA repair [33–39]. Thus, the downregulation of *Yeats4* causes a functional decline of the DNA repair mechanism. Taken together, our findings suggest that Sbno1-mediated regulation of Yeats4 may constitute part of the molecular mechanism underlying IEG regulation.

Although our findings reveal a novel molecular mechanism protecting neuronal genome, a complete overview of DNA repair processes in neurons is needed. The overexpression of Yeats4 in Sbno1 KD neurons only partially rescued neuronal survival. Thus, Yeats4 is an important component protecting genomic DNA regulated by Sbno1.However, it is not the sole factor that functions downstream of Sbno1. Comprehensive analyses, such as ChIP-seq for Sbno1, would provide a complete picture of the downstream molecules of Sbno1 and elucidate the overall molecular function of Sbno1.

Damage to neuronal genome integrity can be implicated in neurodegenerative and neurodevelopmental diseases [11, 40–43]. Mutations in *SBNO1* have been reported in the genomes of neurodevelopmentally affected individuals [11–16]. This study does not elucidate the mechanism of *SBNO1* contributing to neurological diseases. Given that neuronal genome instability has been implicated in neurological diseases, dysfunction of a molecular network in which SBNO1 and YEATS4 are involved may contribute to disease pathogenesis.

## Materials and methods

### Animals

*Sbno1* ^*flox/flox*^; *Nex*-*Cre* (*Sbno1* cKO) mice have been previously described [17]. C57BL/6J mice were obtained from Japan SLC. All animal experiments were performed in accordance with the guidelines of Shiga University of Medical Science. Mice were anesthetized with isoflurane inhalation solution (Pfizer, 219KAT) using NARCOBIT-E (Natsume Seisakusho Co., Ltd) and transcardially perfused with 4% paraformaldehyde in 0.1 M phosphate buffer solution (PBS), and then the brains were dissected for histological analyses.

### Histology

Brain sections were cut to 7 μm thickness. After deparaffinization, the sections were stained using Cresyl Violet solution (Muto Pure Chemicals Co. Ltd.). The brain sections were immunostained as previously described [17]. A TUNEL staining kit (Wako) was used to detect apoptotic cells on the brain sections.

### RNA-seq

Total RNA was extracted from P4 and P7 cerebral cortices using Sepasol RNA I Super G (Nacalai Tesque). cDNA libraries were prepared using ReverTra Ace^®^ qPCR RT Master Mix (Toyobo Inc.) and sequenced using the NextSeq500 platform (Illumina Inc.). The data were analyzed using edgeR in the CLC Genomics Workbench (CLC bio), and the GO DEGs [false discovery rate *p* < 0.05, transcripts per million (TPM) > 1.0] were evaluated using DAVID Bioinformatics Resources (https://david.ncifcrf.gov/). Raw data is uploaded as Supplementary Table S3.

### Microarray analysis

Total RNA was purified from mouse embryonic fibroblasts using the NucleoSpin kit (Takara Bio Inc.). Gene expression profiles were investigated using microarrays (Agilent, SurePrint G3 Mouse GE microarray) and 3D-Gene miRNA Oligo chips (Toray Industries, Inc.). Raw data is uploaded as Supplementary Table S4.

### RT–qPCR

RNA was extracted from the control and *Sbno1* cKO cortices using Sepasol RNA I Super G (Nacalai Tesque). cDNA libraries were prepared using ReverTra Ace^®^ qPCR RT Master Mix (Toyobo). qPCRs were run in a StepOne Plus real-time qPCR system (Applied Biosystems). The primers used are listed in Supplementary Table S2.

### Immunoprecipitation

P0 mouse cerebral cortices were lysed in NP40 lysis buffer (10 mM HEPES [pH 7.6], 250 mM NaCl, 0.1% NP-40, 5 mM EDTA, Protease Inhibitor Cocktail Set III [Millipore]). Protein samples were incubated with 2 μL of the anti-Sbno1 antibody (Abcam ; ab122789) or anti-IgG antibody (CST, #2729) as controls for 2 h at 4 °C. Then, 30 μL of the Dynabeads (Thermo Fisher Scientific) were treated for 1 h at 4 °C, followed by several washes.

### NanoLC–MS/MS system

For sample preparation, proteins were eluted with 0.1 M glycine-HCl (pH 3.0), and the eluate was immediately neutralized with 1 M Tris-HCl (pH 8.5). Recovered proteins were subjected to denaturation, reduction, and alkylation with 8 M urea, 10 mM DTT, and 50 mM IAA. The sample solutions were digested with Lys-C for 3 h, 5-fold diluted with 50 mM ammonium bicarbonate, digested by trypsin overnight, acidified with 0.5% TFA (final concentration) and desalted with C18-StageTips [44].

NanoLC-MS/MS analyses were conducted using a timsTOF Pro mass spectrometer equipped with a nanoElute HPLC (Bruker Daltonics, Bremen, Germany). Peptides were separated on an ODYSSEY column (1.6 µm C18, 120 Å, 75 µm ID, 25 cm; IonOpticks). The 2 µL sample solution flowed at 400 nL/min. The mobile phases consisted of (A) 0.1% formic acid and (B) 0.1% formic acid in 80% acetonitrile. A four-step linear gradient of 4%–34% B in 90 min, 34%–48% B in 15 min, 48%–90% B in 5 min, and 90% B for 10 min was employed. The mass scan range was 100–1700 m/z, and the ion mobility was scanned from 0.6 to 1.6 Vs/cm^2^. The overall scan cycle was 1.16 s and included a single MS scan and 10 PASEF-MS/MS scans [45]. Low-abundance precursor ions below a target intensity value of 20,000 counts were repeatedly selected for PASEF-MS/MS. Active exclusion time was set to 0.4 s.

The raw files were analyzed with PEAKS studio X+ software (Bioinformatics Solutions Inc., Waterloo, ON, CA). Peptides and proteins were identified against UniProtKB/Swiss-prot with a precursor mass tolerance of 20 ppm, a fragment ion mass tolerance of 0.1 Da [46]. Cysteine carbamidomethylation was set as a fixed modification, and methionine oxidation and protein N-terminal acetylation were allowed as variable modifications. The peptide score (−10 lgP) was derived from the *p* value which indicated the statistical significance of the peptide-precursor spectrum match, and the protein score (−10 lgP) was calculated as the weighted sum of the −10 lgP scores of the protein’s supporting peptides. The peptides were sorted by −10 lgP scores in descending order, and a kth-ranked peptide contributed to the weighted sum with a weight of 1/k. Proteins with −10 lgP value of >20 were identified. Label-free quantitation was performed by the PEAKS Q module. Raw data is uploaded as Supplementary Table S5.

### AirID

We constructed pcDNA3.1-AGIA-mSbno1-AirID, with pEF1α-3xHA-Rolr2a and pEF1α-3xHA-Rolr2b plasmids for overexpression in HEK293T cells. The AirID assay was performed following the protocol described by Kido et al. [25].

### Western blotting

Immunoblotting was performed following the ATTO standard protocol. Membranes were blocked with 5% non-fat dry milk in TBST (Tris-buffered saline containing 0.1% Tween-20) for 1 h at room temperature and incubated overnight at 4 °C with the following primary antibodies: anti-Sbno1(original), anti-Yeats4 (aviva, ARP30118_P050), anti-FLAG (Wako, 012-22384), anti-HA (aves, AB_2313511), anti-GFP (aves, #GFP-1020) and anti-β-actin (Proteintech, 81115-1-RR) as a loading control. After washing, membranes were incubated with HRP-conjugated secondary antibodies anti-rabbit IgG-HRP (Merck, MAB201P), anti-mouse IgG-HRP (Proteintech, #SA00001-1) and anti-chicken IgG-HRP (Abcam, ab97135) for 1 h at room temperature. For signal detection, Chemi-Lumi One Super (Detection reagent, Nacalai Tesque) and FUSION (Detector, M&S Instruments Inc.) were used. Original western blot pictures are shown in Supplementary Material file (full images of Western blotting).

### Cleavage under targets and release using nuclease

CUT&RUN was performed following the standard protocol of the kit (86 652, CST). Neuro2a cells were transfected with pCAG-3xHA-m*Sbno1* or pCAG-EGFP (a negative control). Anti-Sbno1 antibody(original) was used for immunoprecipitation.

### Knockdown experiments of Sbno1 and Yeats4

Human embryonic kidney 293 T cells were cultured in 15-cm dishes and transduced with lentiviral constructs (provided by the late Dr. H. Miyoshi and RIKEN BRC). The shRNA sequences are listed in Supplementary Table S1. We constructed gene knockdown (KD) plasmids containing three different short hairpin RNA (shRNA) sequences targeting *Sbno1* or *Yeats4*. To assess the efficacy of the shRNA-mediated KD, we co-transfected the shRNA constructs with plasmids that overexpress Sbno1 or Yeats4 fused to mKate2 fluorescent protein. The efficiency of shRNA constructs was quantified by flowcytometry. The most pronounced decrease in fluorescence intensity was obtained by shSbno1#1 and shYeats4#1 (Fig. S4A).

### Neuronal primary culture

The protocol used for neuronal primary culture is described in Olsen et al. [47]. Approximately 50% of the medium was changed every 2 days. The cells were then fixed in PBS containing 4% PFA for immunocytochemistry.

### Comet assay

A single-cell gel electrophoresis comet assay was performed using a kit (R&D SYSTEMS, INC). Neurons from P0 mouse brains were primarily cultured for 5 days, infected with constructed lentivirus, and collected the next day. DNA was stained with CYGREEN for 30 min.

## Supplementary information

Supplementary Figure 4

Supplementary Figure legend

Supplementary Figure 1

Supplementary Figure 2

Supplementary Figure 3

Supplementary Figure 5

full images of Western blotting

Supplementary Table 1

Supplementary Table 2

Supplementary Table 3

Supplementary Table 4

Supplementary Table 4

## Data Availability

All data are available from the corresponding author upon reasonable request.
