# Supplementary figures and images for: Strawberry notch 1 safeguards neuronal genome via regulation of Yeats4 expression
Source: Cell Death Discov. 2025 Jul 24;11:342. doi: 10.1038/s41420-025-02640-4 (PMC12289961; doi:10.1038/s41420-025-02640-4)

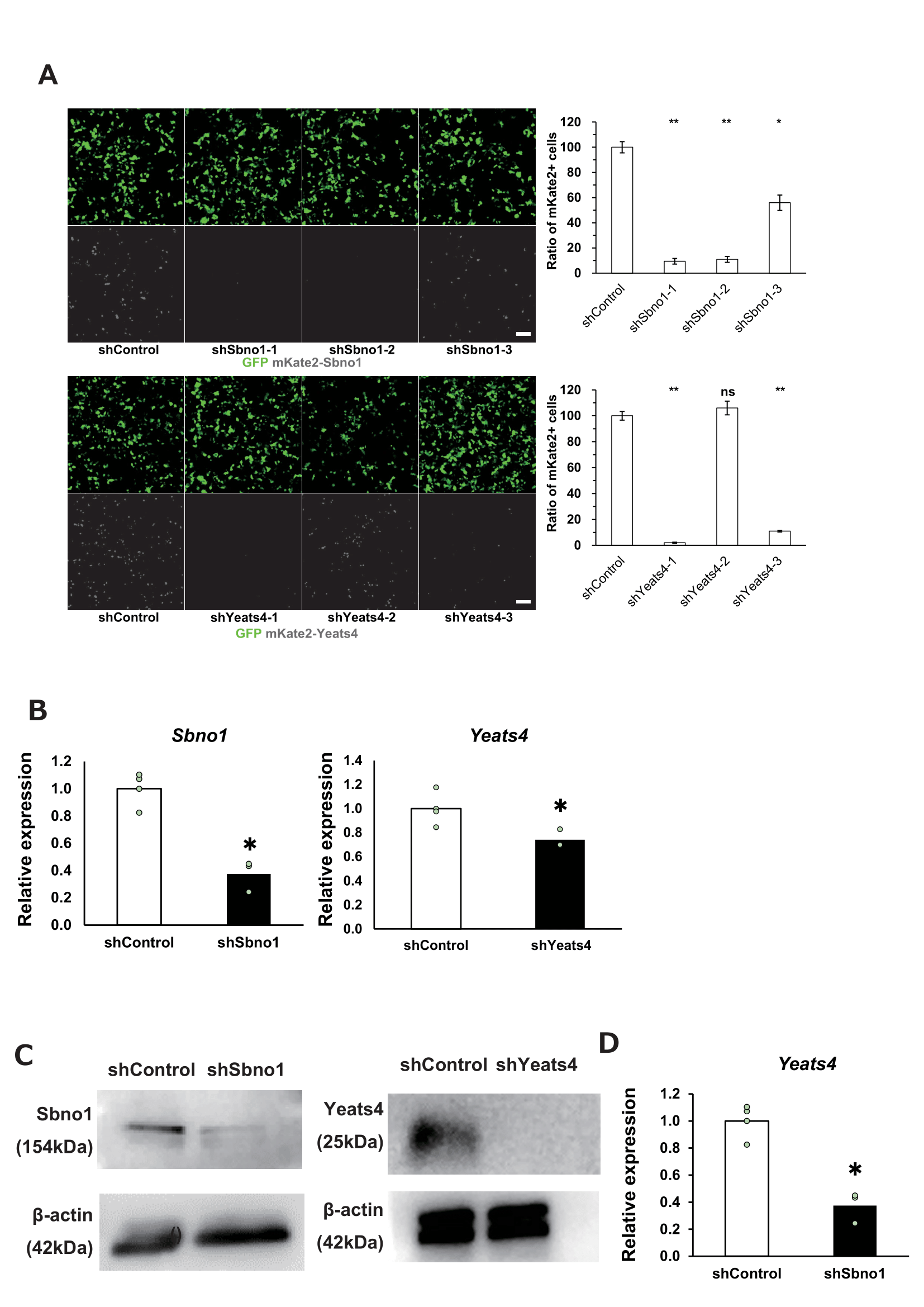

Supplement: Supplementary file 1 — Supplementary Figure 4 [file 41420_2025_2640_MOESM1_ESM.png]

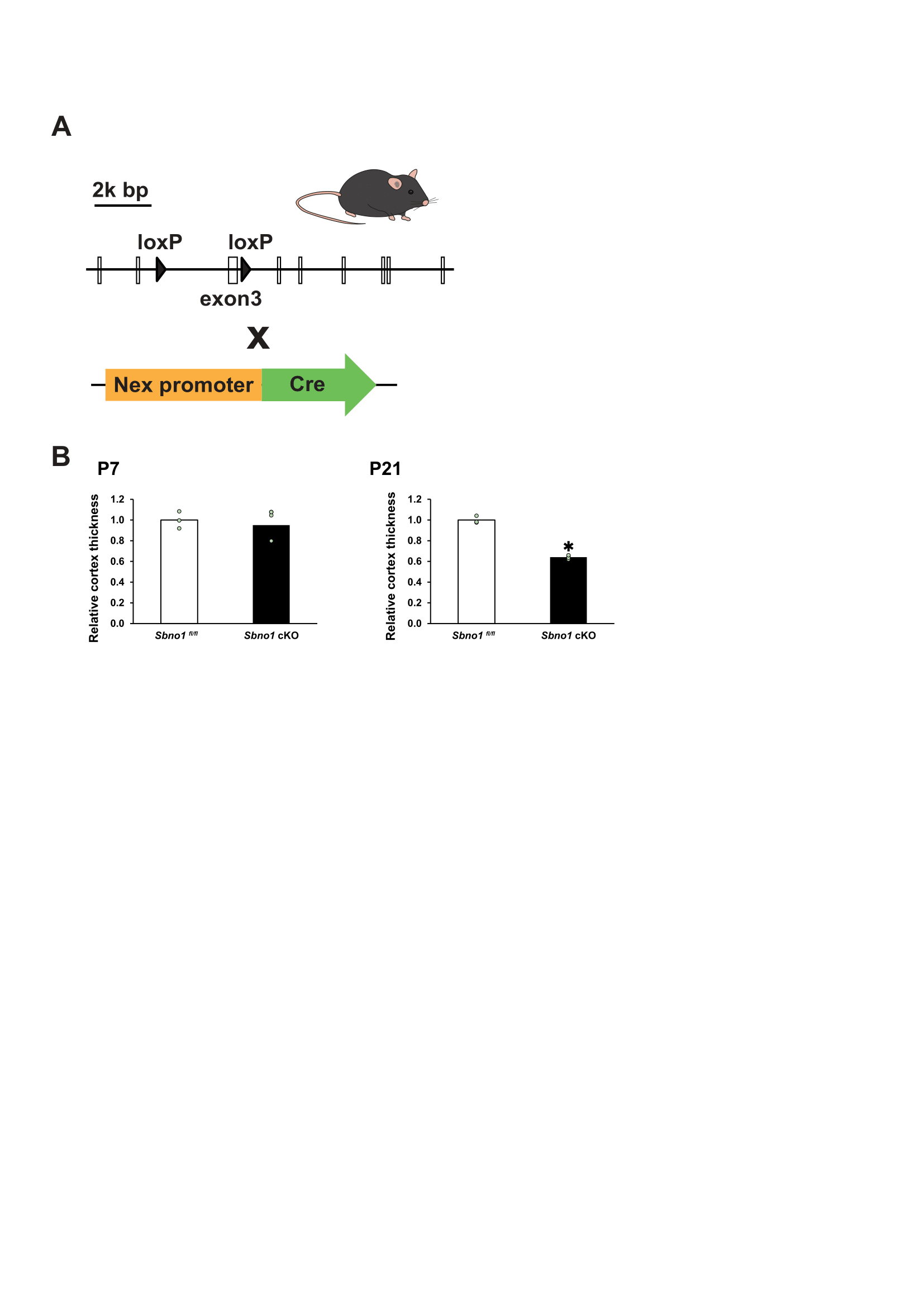

Supplement: Supplementary file 3 — Supplementary Figure 1 [file 41420_2025_2640_MOESM3_ESM.png]

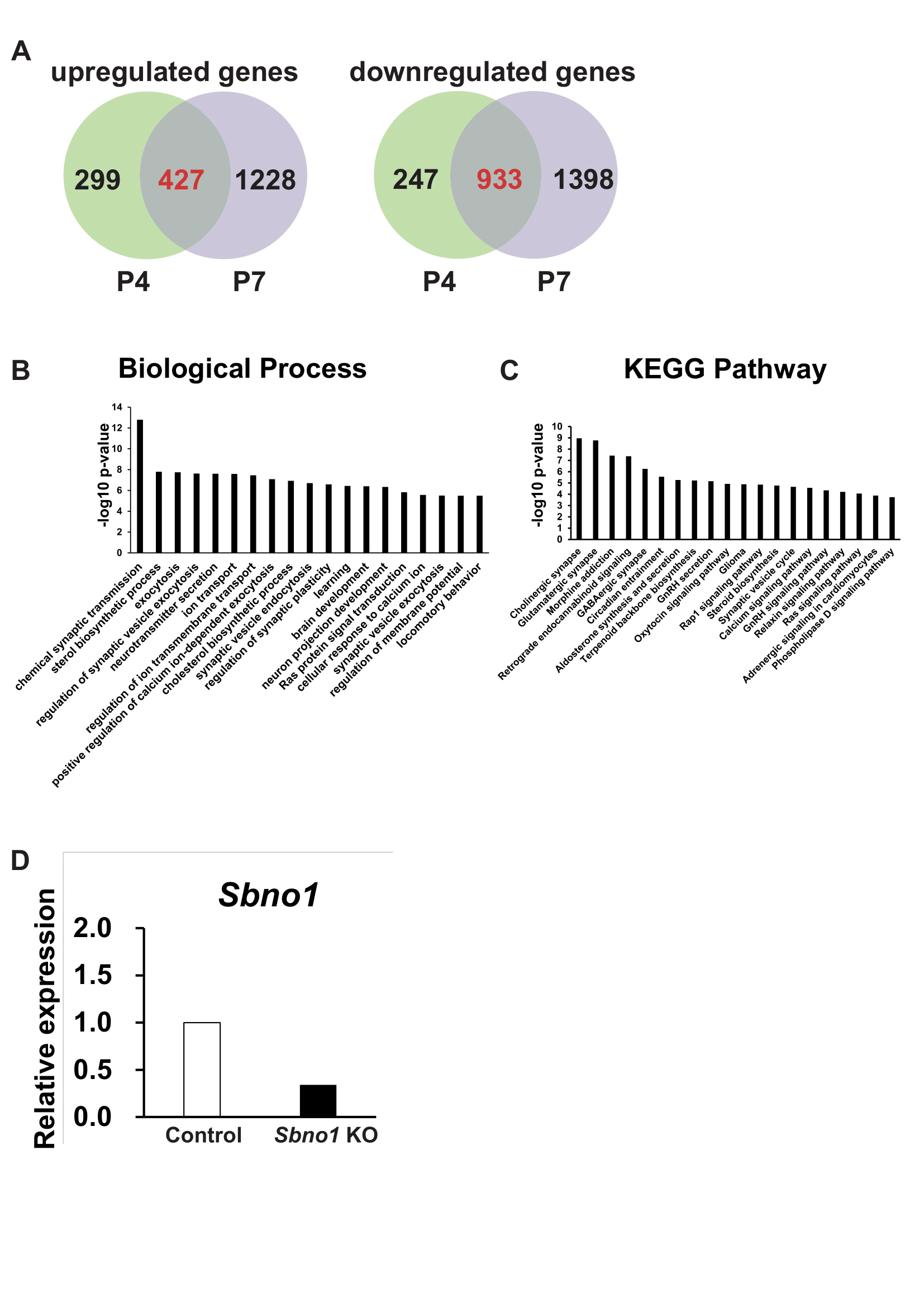

Supplement: Supplementary file 4 — Supplementary Figure 2 [file 41420_2025_2640_MOESM4_ESM.png]

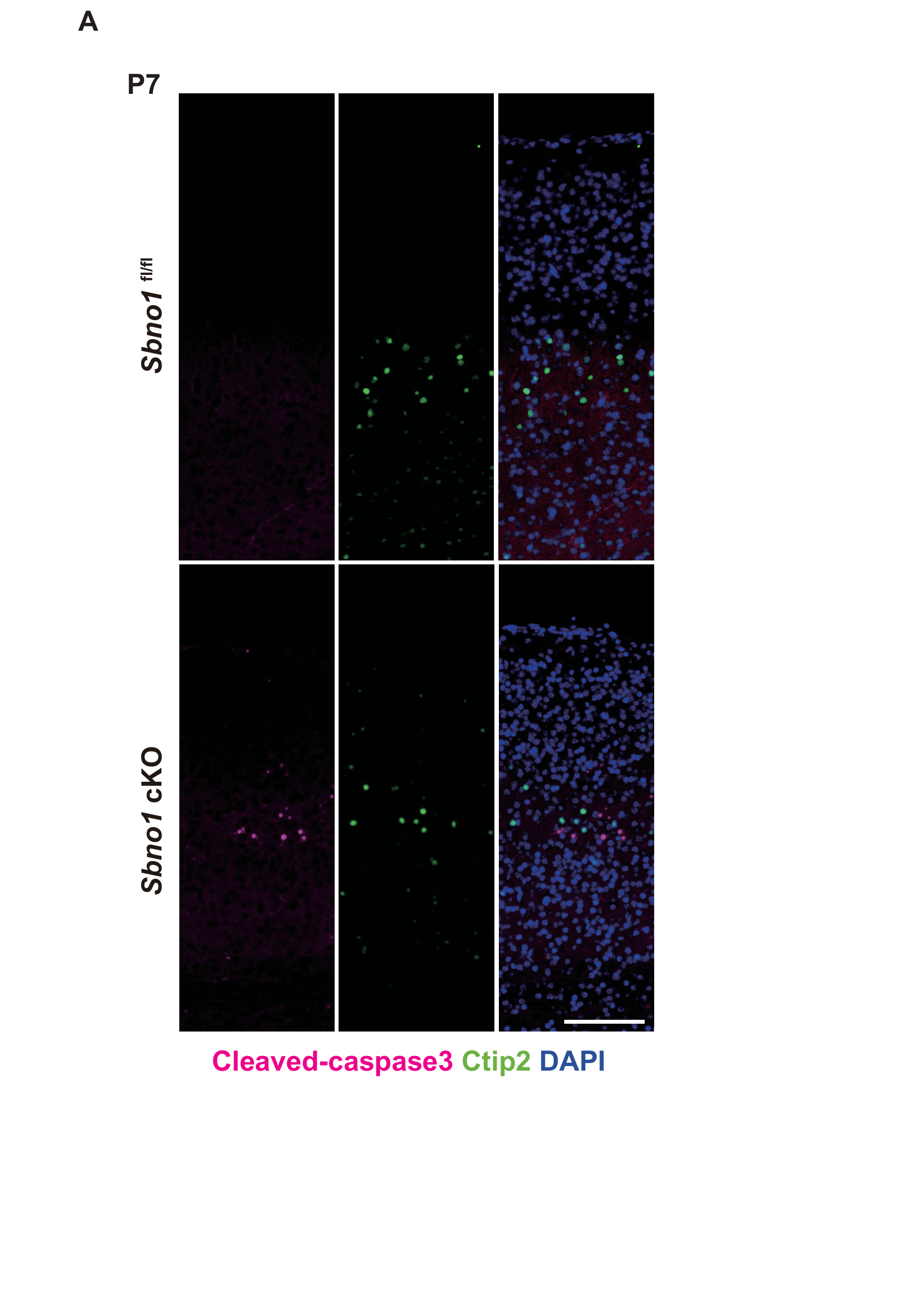

Supplement: Supplementary file 5 — Supplementary Figure 3 [file 41420_2025_2640_MOESM5_ESM.png]

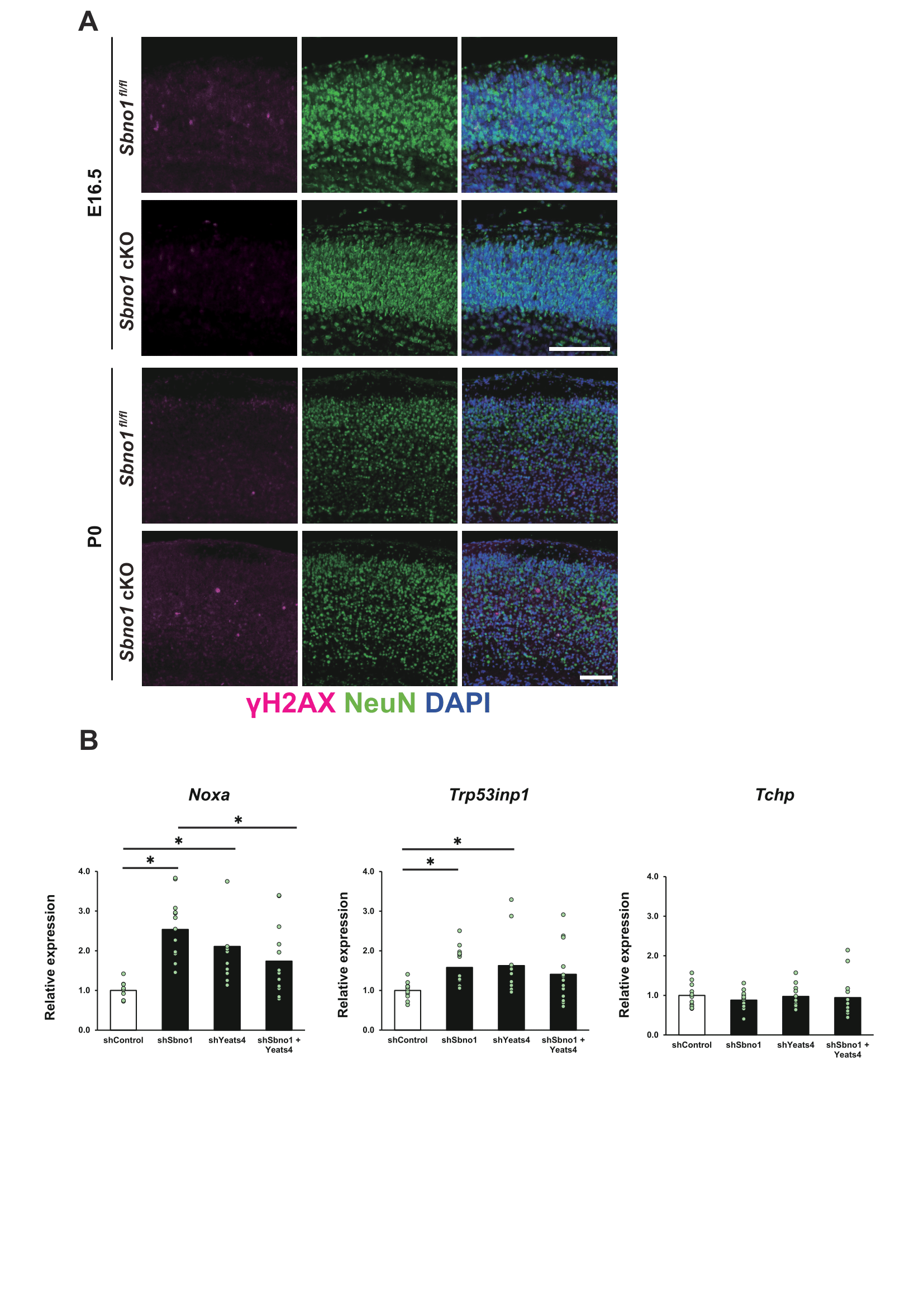

Supplement: Supplementary file 6 — Supplementary Figure 5 [file 41420_2025_2640_MOESM6_ESM.png]
