# Supplementary material for: Strawberry notch 1 safeguards neuronal genome via regulation of Yeats4 expression
Source: Cell Death Discov. 2025 Jul 24;11:342. doi: 10.1038/s41420-025-02640-4 (PMC12289961; doi:10.1038/s41420-025-02640-4)
Supplement: Supplementary file 2 — Supplementary Figure legend [file 41420_2025_2640_MOESM2_ESM.pdf]

Supplementary Figure legends

Fig. S1. (A) Schematic illustration of *Sbno1* cKO mice. The third exon of *Sbno1* is flanked by two loxP sequences. Neurons-specific Cre recombinase expression is made by *Nex* promoter. (B) Quantitative comparisons of the thickness of the primary somatosensory cortices between control and *Sbno1* cKO mice. Statistical significance was determined by two-tailed Welch's t-tests; $n = 3$, $p < 0.05$.

Fig. S2. (A) Venn diagram of upregulated and down regulated genes by *Sbno1* cKO shared by P4 and P7 cortices according to results of RNA-seq. (B) GO enrichment analysis of differentially expressed genes shared by P4 and P7 cortices. GO biological processes were obtained by DAVID analysis. (C) GO enrichment analysis of differentially expressed genes shared by P4 and P7 cortices. KEGG pathway database was analyzed by DAVID. (D) Confirmation of reduction in *Sbno1* expression by tamoxifen treatment of $Sbno1^{fl/fl}$*; CAG-CreEstr* MEFs by qPCR.

Fig. S3. Prominent of increase of apoptosis (CC-3 expression) in layer 5 (marked by Ctip2 expression) of *Sbno1* cKO at P7. Scale bar = 100 μm.

Fig. S4. (A) Knockdown efficiency by shRNA plasmids examined by reduction of $mKate2^{+}$ cell. Results are shown as the mean ± S.E.M. (B) Confirmation of effective shRNA-mediated knockdown as assessed by RT-qPCR. Results are quantitatively shown as the mean ± S.E.M. Left panel : shControl : $n = 4$, shSbno1 : $n = 3$. Right panel : shControl : $n = 3$, shYeats4 : $n = 3$. $p < 0.05$. (C) Knockdown of endogenously expressed Sbno1 or Yeats protein in cultured cortical neurons examined by Western blotting. β-Actin

is a loading control. (D) Reduction of Yeats4 expression by Sbno1 knockdown by shRNA. He results were examined quantitatively by qPCR. Results are shown as the mean ± S.E.M. shControl : $n = 4$, shSbno1 : $n = 3$. $p < 0.05$.

Fig. S5. (A) DSBs marked by γH2AX expression (red) in neurons marked by NeuN (green) in *Sbno1* cKO cortex are comparable to that in control at E16.5 and increased at P0. Scale bar: 100 μm. The sections were counterstained by DAPI. Scale bar = 100 μm. (B) Expressions of apoptosis-related factors were examined in primary neuron cultures transfected with shRNA-control, shRNA-Sbno1, shRNA-Yeats4, or sh-Sbno1 and Yeats4 overexpression (rescue experiment) plasmids. Statistical significance of results of qPCR was determined by two-tailed Welch's t-tests; shControl : $n = 11$, shSbno1 : $n = 13$, shYeats4 : $n = 11$, shSbno1+Yeats4 : $n = 13$ *$p < 0.05$.
