# Supplementary material for: Strawberry notch 1 safeguards neuronal genome via regulation of Yeats4 expression
Source: Cell Death Discov. 2025 Jul 24;11:342. doi: 10.1038/s41420-025-02640-4 (PMC12289961; doi:10.1038/s41420-025-02640-4)
Supplement: Supplementary file 7 — full images of Western blotting [file 41420_2025_2640_MOESM7_ESM.pdf]

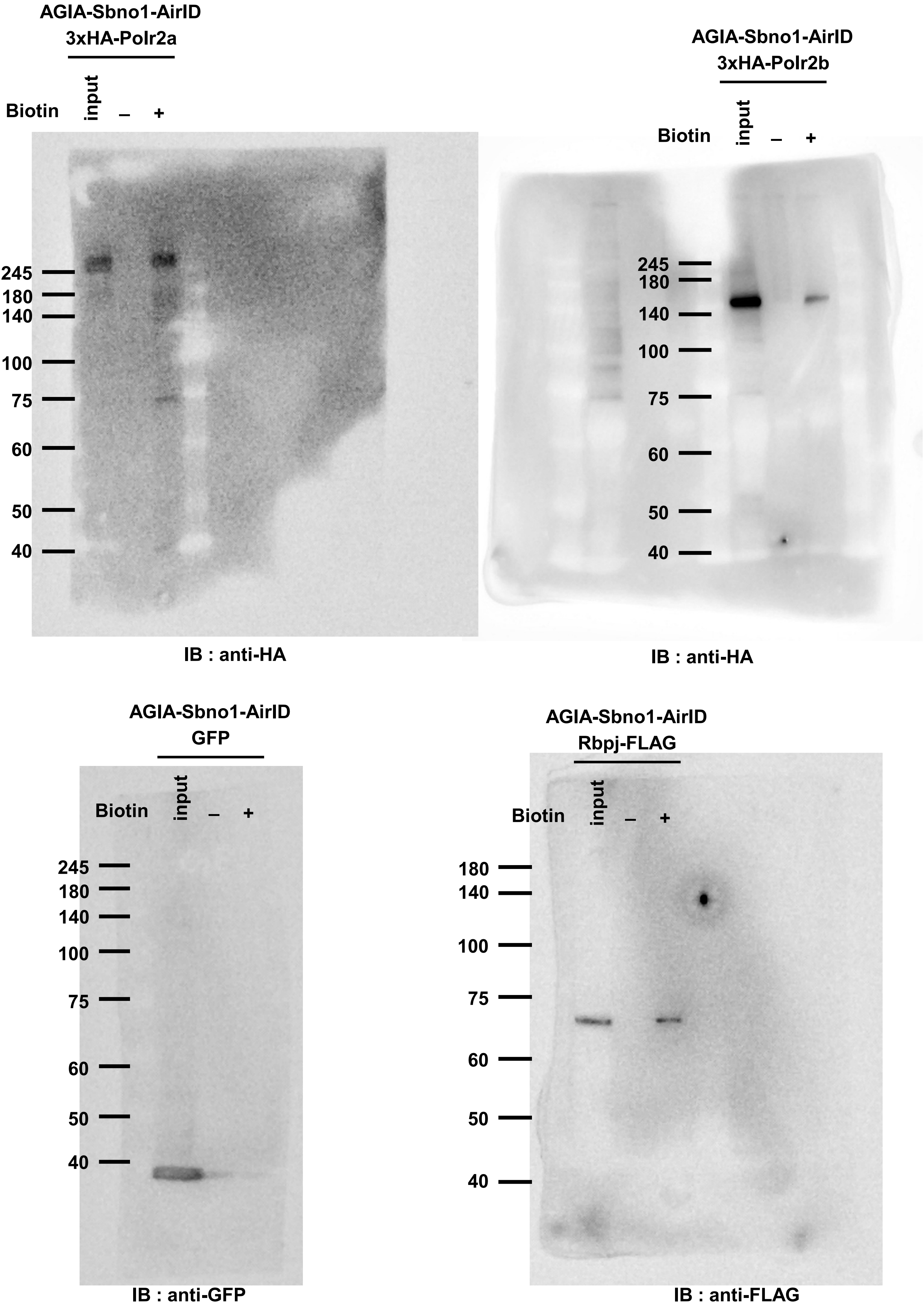
AGIA-Sbno1-AirID
3xHA-Polr2a
Biotin input – +
245
180
140
100
75
60
50
40
IB : anti-HA
AGIA-Sbno1-AirID
3xHA-Polr2b
Biotin input – +
245
180
140
100
75
60
50
40
IB : anti-HA
AGIA-Sbno1-AirID
GFP
Biotin input – +
245
180
140
100
75
60
50
40
IB : anti-GFP
AGIA-Sbno1-AirID
Rbpj-FLAG
Biotin input – +
180
140
100
75
60
50
40
IB : anti-FLAG

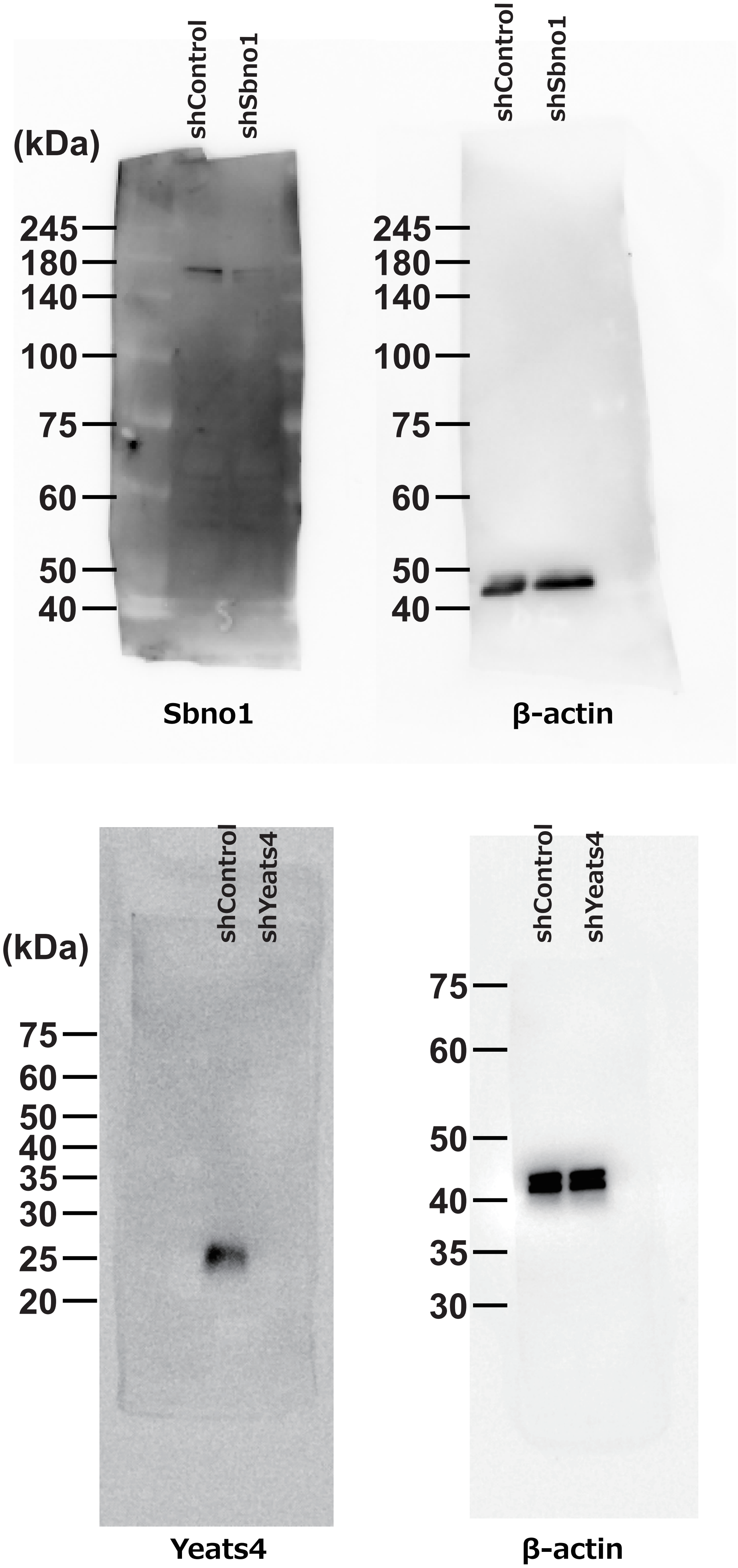
shControl
shSbno1
(kDa)
245
180
140
100
75
60
50
40
Sbno1
shControl
shSbno1
245
180
140
100
75
60
50
40
β-actin
shControl
shYeats4
(kDa)
75
60
50
40
35
30
25
20
Yeats4
shControl
shYeats4
75
60
50
40
35
30
β-actin
